# An extinct deep-snouted *Alligator* species from the Quaternary of Thailand and comments on the evolution of crushing dentition in alligatorids

**DOI:** 10.1038/s41598-023-36559-6

**Published:** 2023-07-13

**Authors:** Gustavo Darlim, Kantapon Suraprasit, Yaowalak Chaimanee, Pannipa Tian, Chotima Yamee, Mana Rugbumrung, Adulwit Kaweera, Márton Rabi

**Affiliations:** 1 Department of Geosciences, Eberhard Karls Universität Tübingen, Hölderlinstraße 12, 72074 Tübingen, Germany; 2 Department of Geology, Faculty of Science, Center of Excellence in Morphology of Earth Surface and Advanced Geohazards in Southeast Asia (MESA CE), Chulalongkorn University, Bangkok, 10330 Thailand; 3 Laboratory PALEVOPRIM, UMR 7262 CNRS, University of Poitiers, 6 Rue Michel Brunet, 86073 Poitiers Cedex 9, France; 4 Department of Mineral Resources, Rama VI Road, Bangkok, 10400 Thailand; 5 Central Natural Science Collections, Martin-Luther University Halle-Wittenberg, 06108 Halle (Saale), Germany

**Keywords:** Palaeontology, Taxonomy

## Abstract

Fossil *Alligator* remains from Asia are critical for tracing the enigmatic evolutionary origin of the Chinese alligator, *Alligator sinensis*, the only living representative of Alligatoridae outside the New World. The Asian fossil record is extremely scarce and it remains unknown whether *A. sinensis* is an anagenetic lineage or alternatively, extinct divergent species were once present. We provide a detailed comparative description of a morphologically highly distinct *Alligator* skull from the Quaternary of Thailand. Several autapomorphic characters warrant the designation of a new species. *Alligator munensis* sp. nov. shares obvious derived features with *A. sinensis* but autapomorphies imply a cladogenetic split, possibly driven by the uplift of the southeastern Tibetan plateau. The presence of enlarged posterior alveoli in *Alligator munensis* is most consistent with a reversal to the alligatorine ancestral condition of having crushing dentition, a morphology strikingly absent among living alligatorids. Crushing dentition has been previously considered to indicate an ecological specialisation in early alligatorines that was subsequently lost in *Alligator* spp*.* However, we argue that there is yet no evidence for crushing dentition reflecting an adaptation for a narrower niche, while opportunistic feeding, including seasonal utilisation of hard-shelled preys, is a reasonable alternative interpretation of its function.

## Introduction

The Chinese alligator, *Alligator sinensis*^1^ is the only extant representative of Alligatoridae (the crown-group of caimans and alligators) outside the Americas. An early divergence age as estimated by molecular clocks from its phylogenetically closest living relative, the North American *Alligator mississippiensis*^2^ (Eocene-early Oligocene^3^) and a poorly sampled Asian fossil record obscures the origin of the Chinese alligator lineage. In particular, the timing and climatic context of *Alligator* dispersal from North America to Asia^3–8^ is poorly constrained.

Owing to a preservational and research bias of the *Alligator* fossil record towards North America (see Stout^9^, and Hastings et al.^10^ for a review), description of fossil material from Asia is essential and much needed comparative morphological data. Previously published *Alligator* fossil material from Asia include an articulated skeleton of *A. luicus*^11^ from the Miocene of China; an altirostral short-snouted skull referred to *A*. cf. *sinensis* from the late Miocene/Pleistocene of Thailand^12^; fragmentary remains of *A. sinensis* from the Pliocene of Japan^13^, and a near-complete skull from the Pleistocene of Taiwan referred to *A. sinensis*^6^.

The subject of this contribution is the DMR-BSL2011-2 *A*. cf. *sinensis* skull from northeastern Thailand preliminarily reported by Claude et al.^12^. Claude et al.^12^ noted similarities with *A. sinensis* as well as its distinctly robust and short snout, but the need for further preparation of the fossil precluded a detailed description at the time. Nevertheless, the occurrence in Thailand considerably expanded the previously known distribution of *Alligator* in Asia, suggesting complex paleobiogeographic history^12^.

Following preparation of DMR-BSL2011-2, we present a comparative description utilising CT-scan imaging data and demonstrate that its highly distinct morphology warrants naming a new species and at the same time several shared several derived characteristics suggest close relationship to *Alligator sinensis*. This new species highlights previously unsampled comparative morphological data for a future comprehensive phylogenetic study of *Alligator* species, critical for reconstructing the biogeographical history of the clade. In addition, we discuss the evolutionary implications of the inferred enlarged posterior dentition of the new species.

### Geological settings

**Figure 1:**
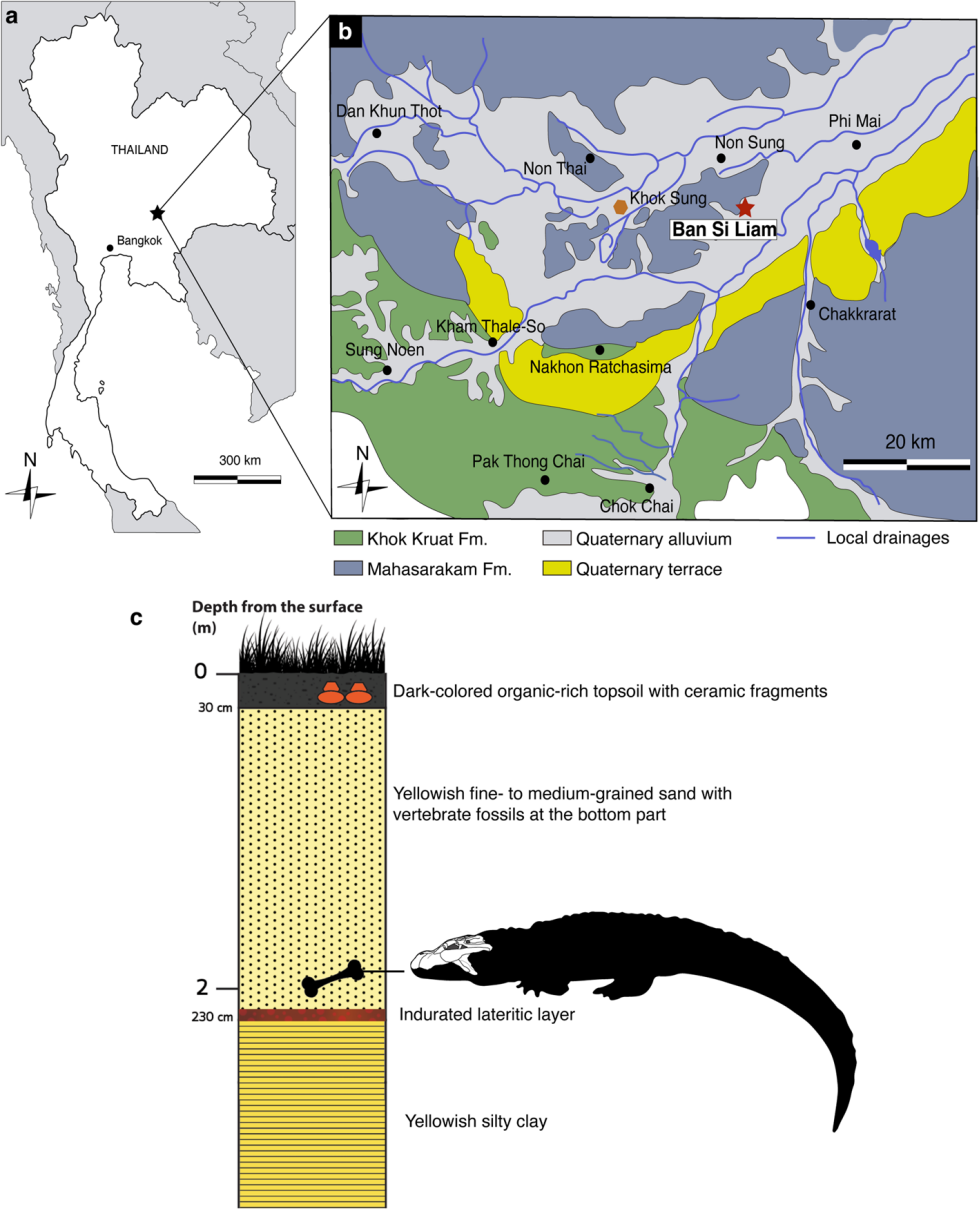
Locality of the *Alligator munensis* sp. nov. holotype (DMR-BSL-2011–2). (**a**) schematic drawing of the map of Thailand. (**b**) geological map of Nakhon Ratchasima province modified after Suraprasit et al.^20^. Red star indicates the type locality of *Alligator munensis* sp. nov. Orange polygon indicates Quaternary fossil site of Khok Sung; (**c**) stratigraphic profile of the fossil site of Ban Si Liam in Non Sung district (Nakhon Ratchasima) showing the level where *Alligator munensis* sp. nov. was found as indicated by the bone and alligator silhouette.

The fossil site (UTM coordinates: 102°15′02.4″ E, 15°08′33.1″ N) is located at Ban Si Liam, Non Sung district, Nakhon Ratchasima Province in northeastern Thailand (Fig. 1a,b). In 2005, the square-shaped pond with an area of 8 m long × 8.4 m wide × 2 m deep was dug out by the villagers and yielded some vertebrate fossils (see Supplementary Information). Regarding the stratigraphic profile of Ban Si Liam (Fig. 1c), the dark-colored topsoil is 30 cm in thickness and organic-rich in content, underlain by yellowish medium- to fine-grained sands with the thickness of 2 m (Supplementary Fig. 1). Some fragments of pottery and ceramics were collected from the topsoil but vertebrate fossils (nine specimens) were only found from the yellowish sandy layer that overlies a thin layer of indurated iron oxide (10 cm thick), followed by the yellowish clay at the lowermost part of the pond. Three reptile fossils included a fragment of a turtle carapace (DMR-BSL2011-1) and a nearly complete cranium of an alligator (DMR-BSL2011-2), both of which have been previously reported by Claude et al.^12^, as well as a crocodylian vertebra (DMR-BSL2011-3; Supplementary Fig. 2).

In addition to the alligator skull described in this study, fossils of two mammalian species collected from the same layer were identified as belonging to a wild water buffalo (*Bubalus arnee*) and a sambar deer (*Rusa unicolor*) (Supplementary Fig. 3) based on the comparisons of morphological features and dimensions with extant comparative specimens and fossils recovered nearby (i.e. the late Middle Pleistocene fauna from Khok Sung^14^). We find no evidence for the presence of giraffids (otherwise known from the Late Miocene of Thailand) in contrast to the report of Claude et al.^12^. The presence of *Bubalus arnee* and *Rusa unicolor*, on the other hand suggests a younger and narrower age range than the previously proposed Late Miocene to Pleistocene^12^ as these taxa are typical for late Middle Pleistocene faunas of Thailand like that of Tham Wiman Nakin (dated to > 169 ka^15–17^) or Khok Sung (dated to either 217 or 130 ka^14,18^). Moreover, the stratigraphic position of the fossiliferous layer at Ban Si Liam is quite shallow (around 2 m below the surface, Fig. 1c) compared to other Late Miocene deposits along the Mun River systems (i.e. around 10 to 20 m deep in Tha Chang sandpits^19^) (see Supplementary Information for detailed geological settings). A Holocene age of the locality cannot be excluded at the moment.

### Institutional abbreviations

AMNH–American Museum of Natural History, New York, New York, USA; DMR–Department of Mineral Resources, Bangkok, Thailand; FMNH–Field Museum of Natural History, Chicago, Illinois, USA; IRScNB–Institut Royal des Sciences Naturelles de Belgique, Brussels, Belgium; MCZ–Museum of Comparative Zoology, Harvard University, Cambridge, Massachusetts, USA; SNSB–Staatliche Naturwissenschaftliche Sammlungen Bayerns, Munich, Germany; SZ–Museum der Universität Tübingen, Zoologisches Schausammlung, Tübingen, Germany; YPM–PU–Princeton University collection housed at Peabody Museum, New Haven, Connecticut, USA.

## Results

### Systematic palaeontology

E﻿usuchia﻿ Huxley, 1875^21^ sensu Brochu, 2003^22^

Crocodylia Gmelin, 1789^23^ sensu Benton & Clark, 1988^24^

Alligatoroidea Gray, 1844^25^ sensu Brochu, 2003^22^

Globidonta Brochu, 1999^4^

Alligatoridae Cuvier, 1807^26^ sensu Brochu, 2003^22^

Alligatorinae Kälin, 1940^27^

*Alligator* Cuvier, 1807^26^ sensu Brochu, 1999^4^

**Figure 2:**
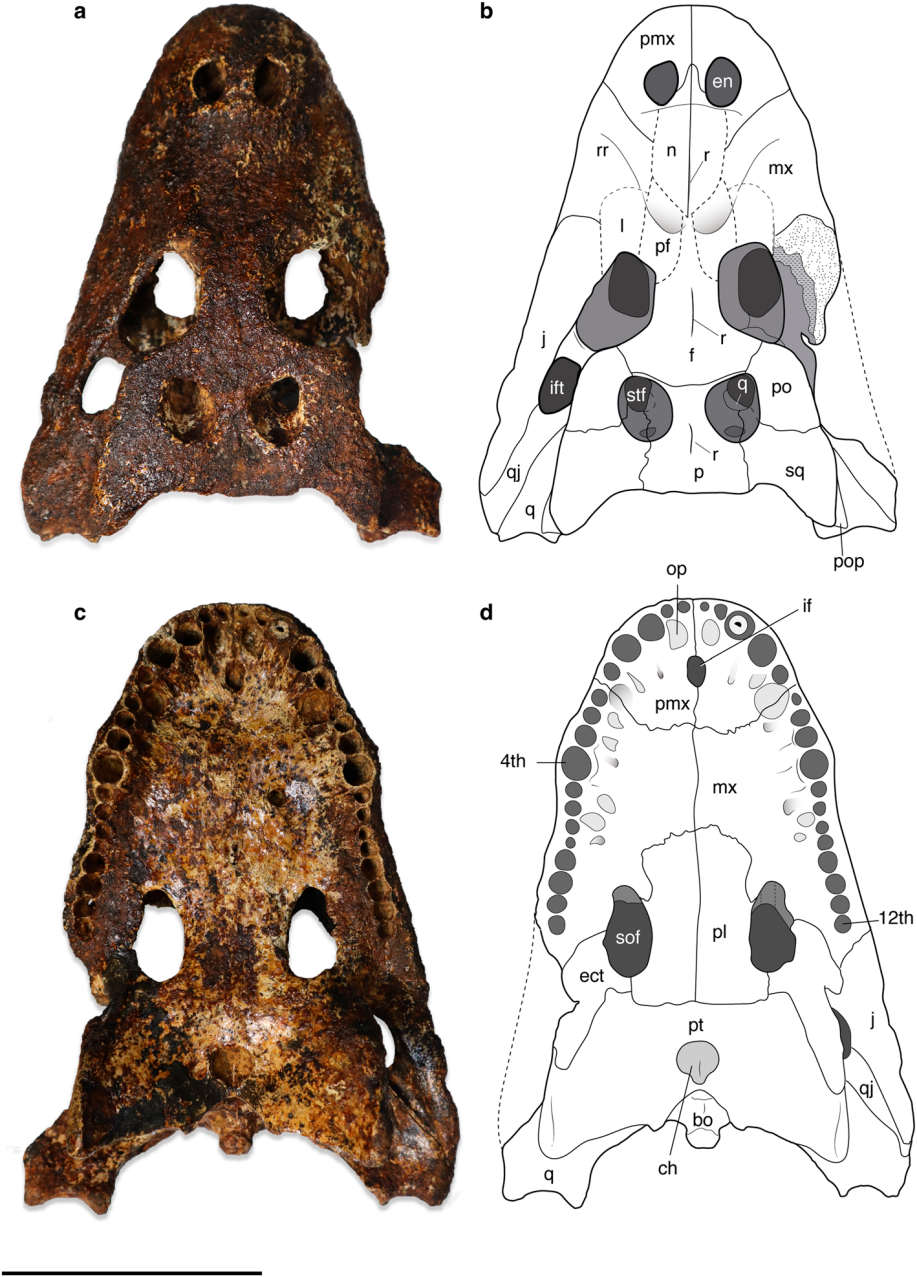
Skull of *Alligator munensis* sp. nov., holotype (DMR-BSL-2011–2). Photo and schematic drawing in (**a**,**b**) dorsal, and (**c**,**d**) ventral views, respectively. *bo* basioccipital, *ch* choana, *ect* ectopterygoid, *en* external nares; *f* frontal, *if* incisive foramen, *itf* infratemporal fenestra, *j* jugal, *l* lacrimal, *mx* maxilla, *n* nasal, *p* parietal, *pf* prefrontal, *pl* palatine, *pt* pterygoid, *pmx* premaxilla, *op* occlusal pit, *po* postorbital, *pop* paroccipital process, *q* quadrate, *qj* quadratojugal, *r* ridge, *rr* rostral ridge, *sof* suborbital fenestra, *sq* squamosal, *stf* supratemporal fenestra, 4th, fourth maxillary alveolus; 12th, twelfth maxillary alveolus. Scale bar: 10 cm.

**Figure 3:**
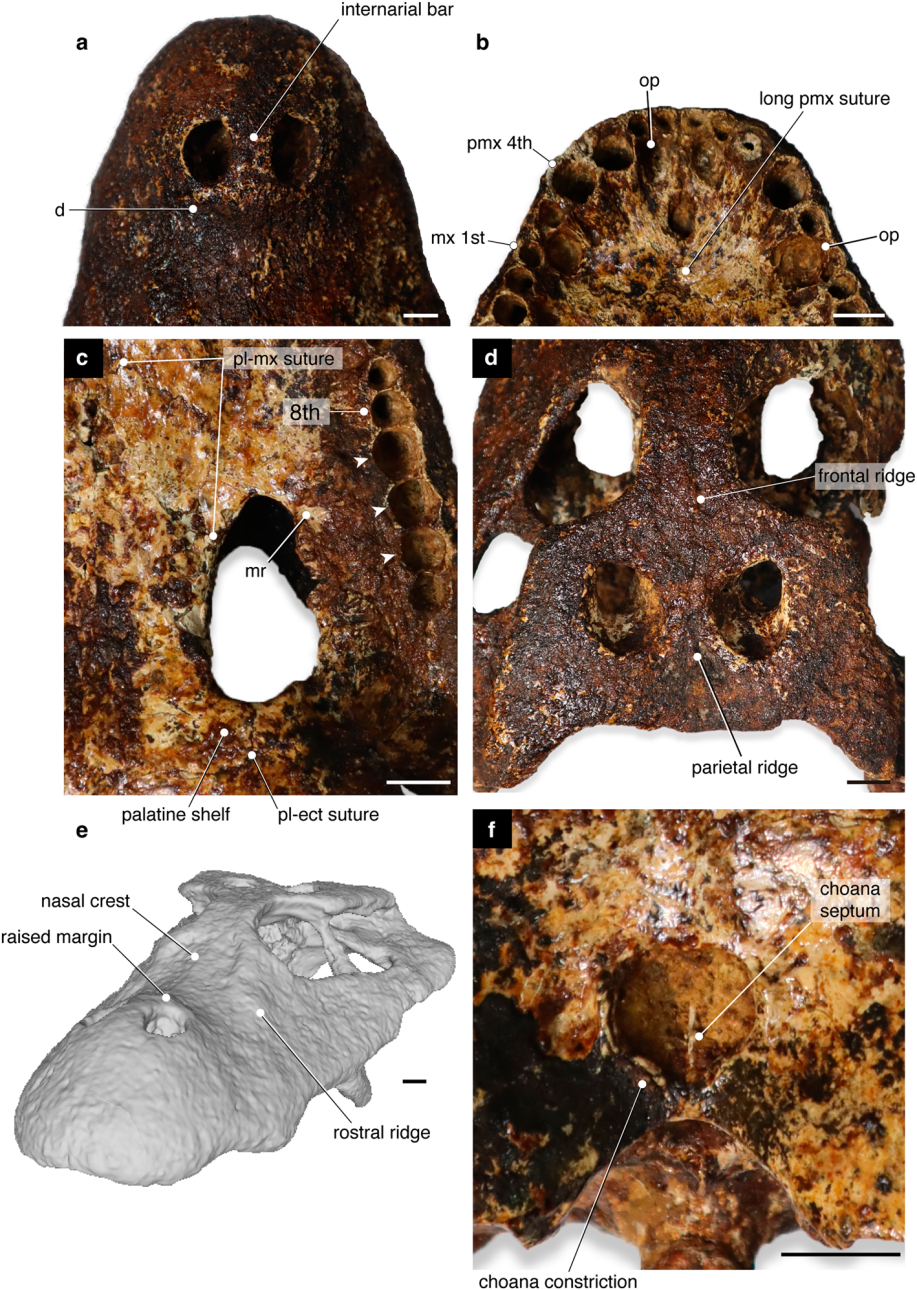
Details of the skull of *Alligator munensis* sp. nov. holotype (DMR-BSL-2011–2). (**a**) Anterior portion of the snout in dorsal view; (**b**) premaxillae in ventral view; (**c**) detail of the bone elements composing the suborbital fenestra; (**d**) skull table in dorsal view; (**e**) digitally reconstructed skull in oblique view; (**f**) choana White arrows indicate enlarged maxillary alveoli. *d*, depression; *ect*, ectopterygoid; *mr*, maxillary rugosity; *mx*, maxilla; *mx 1st*, first maxillary alveolus; *op*, occlusal pit; *pl*, palatine; *pmx*, premaxilla; *pmx 4th*, fourth premaxillary alveolus; *8th*, eighth maxillary alveolus. Scale bars: 1 cm.

**Figure 4:**
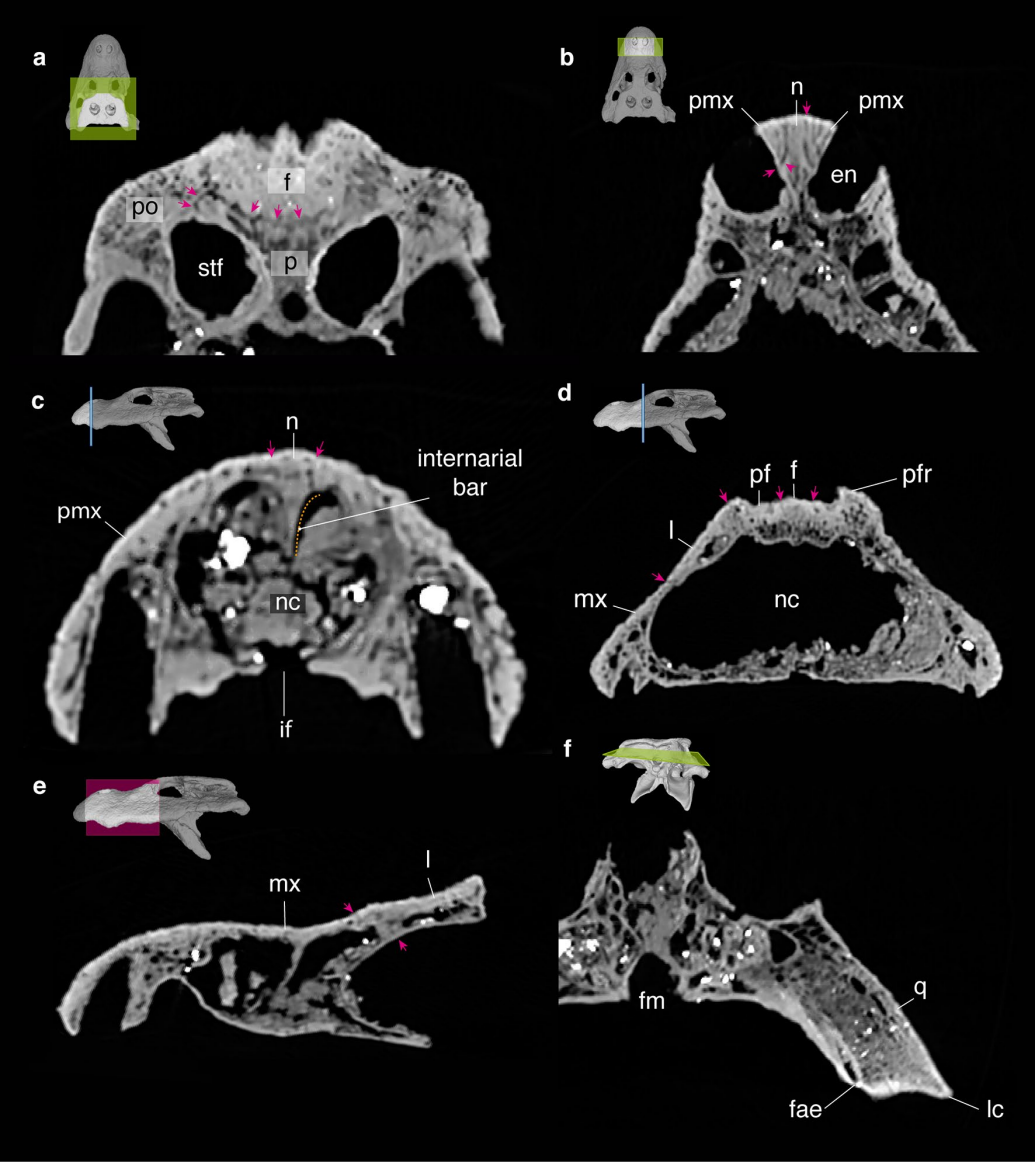
Selection of CT scan image stacks of *Alligator munensis* sp. nov. holotype (DMR-BSL-2011-2). Each figure component (**a**–**f**) is followed by the digitally reconstructed skull of *A. munensis* highlighting the area of interest. (**a**) axial cut of the skull table showing the fronto-parietal suture; (**b**) axial and (**c**) coronal cuts of the snout region showing the composition and morphology of the internarial bar; (**d**) coronal cut of the posterior portion of the nasal cavity; (**e**) sagittal cut of the antorbital region of the skull; (**f**) axial cut of the posterior position of the skull. Pink arrows indicate sutures between the bone elements. Dashed orange line indicates the contour of the internarial bar. *en* external nares, *f* frontal, *fae* foramen äerum, *fm* foramen magnum, *if* incisive foramen, *l* lacrimal, *lc* lateral quadrate hemicondyle, *mx* maxila, *n* nasal, *nc* nasal cavity, *p* parietal, *pf* prefrontal, *pfr* prefrontal ridge, *pmx* premaxilla; *po* postorbital, *q* quadrate, *stf* supratemporal fenestra.

**Figure 5:**
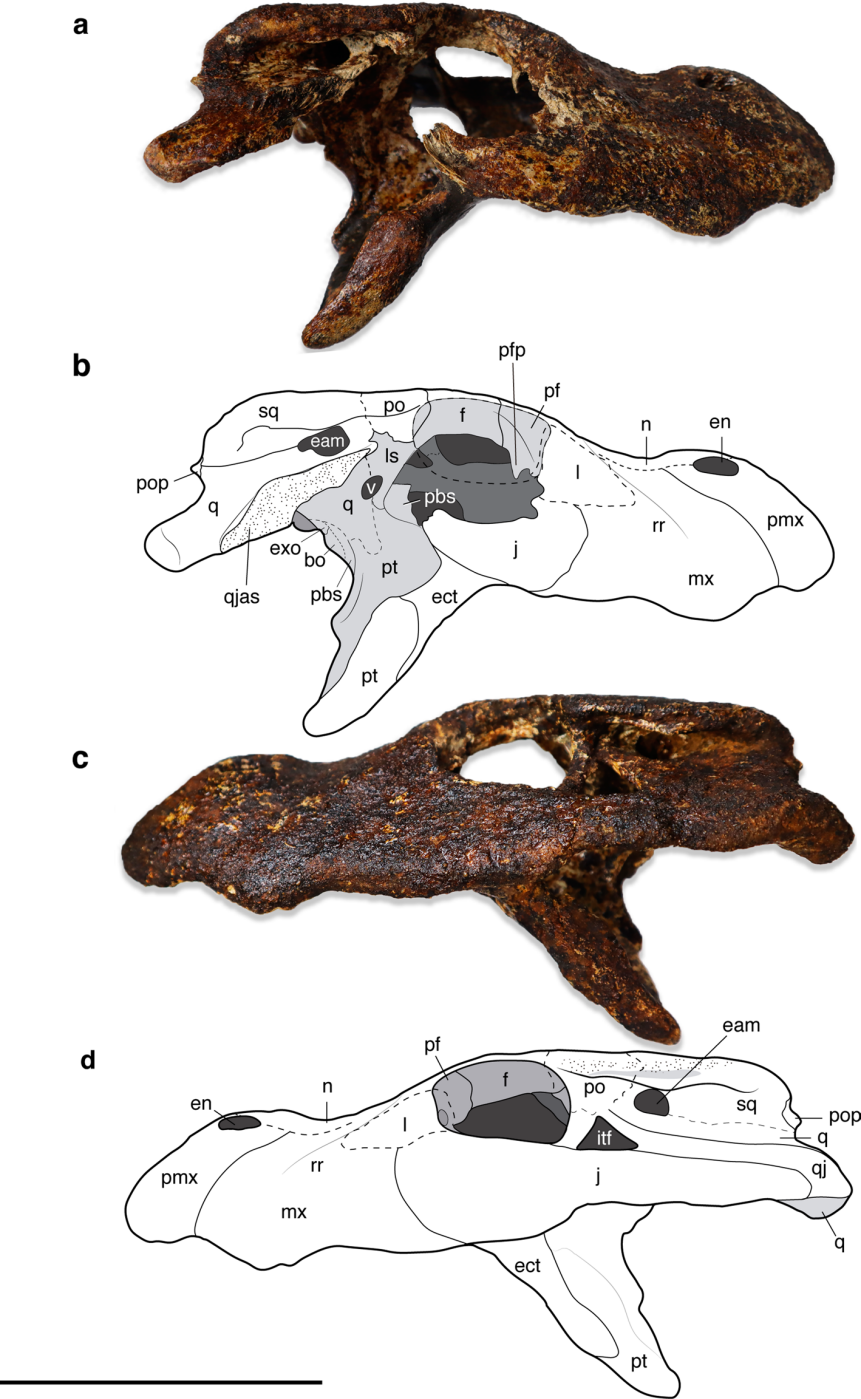
Skull of *Alligator munensis* sp. nov. holotype (DMR-BSL-2011–2) and schematic drawing in right (**a**,**b**) and left (**c**,**d**) lateral views, respectively. *bo* basioccipital, *eam* external auditory meatus, *ect* ectopterygoid, *en* external nares, *exo* exoccipital, *f* frontal, *itf* infratemporal fenestra, *j* jugal, *l* lacrimal, *ls* laterosphenoid, *mx* maxilla, *n* nasal, *pbs* parabasisphenoid, *pf* prefrontal, *pfp* prefrontal pillar, *pmx* premaxilla, *po* postorbital, *pop* paroccipital process, *pt* pterygoid, *q* quadrate, *qj* quadratojugal; *qjas* quadratojugal articular surface, *rr* rostral ridge, *sq* squamosal; *V* foramen for the trigeminal nerve. Scale bar: 10 cm.

**Figure 6:**
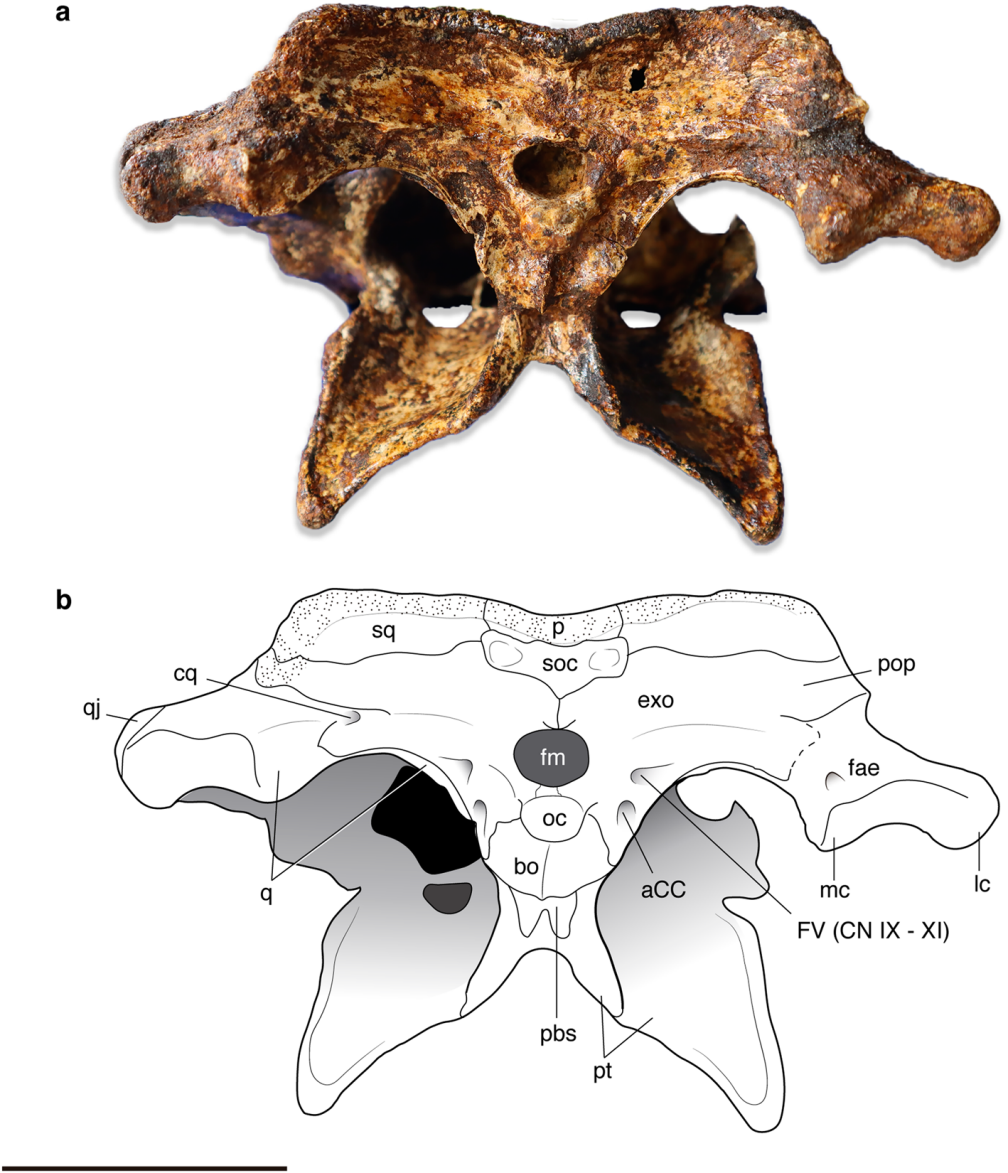
Skull of *Alligator munensis* sp. nov. holotype (DMR-BSL-2011–2) (**a**) photograph and (**b**) schematic drawing in occipital view. *aCC* foramen for cerebral carotid artery, *bo* basioccipital, *cq* cranioquadrate passage, *exo* exoccipital, *fae* foramen aërum, *fm* foramen magnum; FV (CN IX–XI), foramen vagi for the passage of cranial nerves IX–XI; *lc* lateral quadrate hemicondyle, *mc* medial quadrate hemicondyle, *oc* occipital condyle, *p* parietal, *pbs* parabasisphenoid, *pop* paroccipital process, *pt* pterygoid, *q* quadrate, *qj* quadratojugal, *soc* supraoccipital, *sq* squamosal. Scale bar: 5 cm.

**Figure 7:**
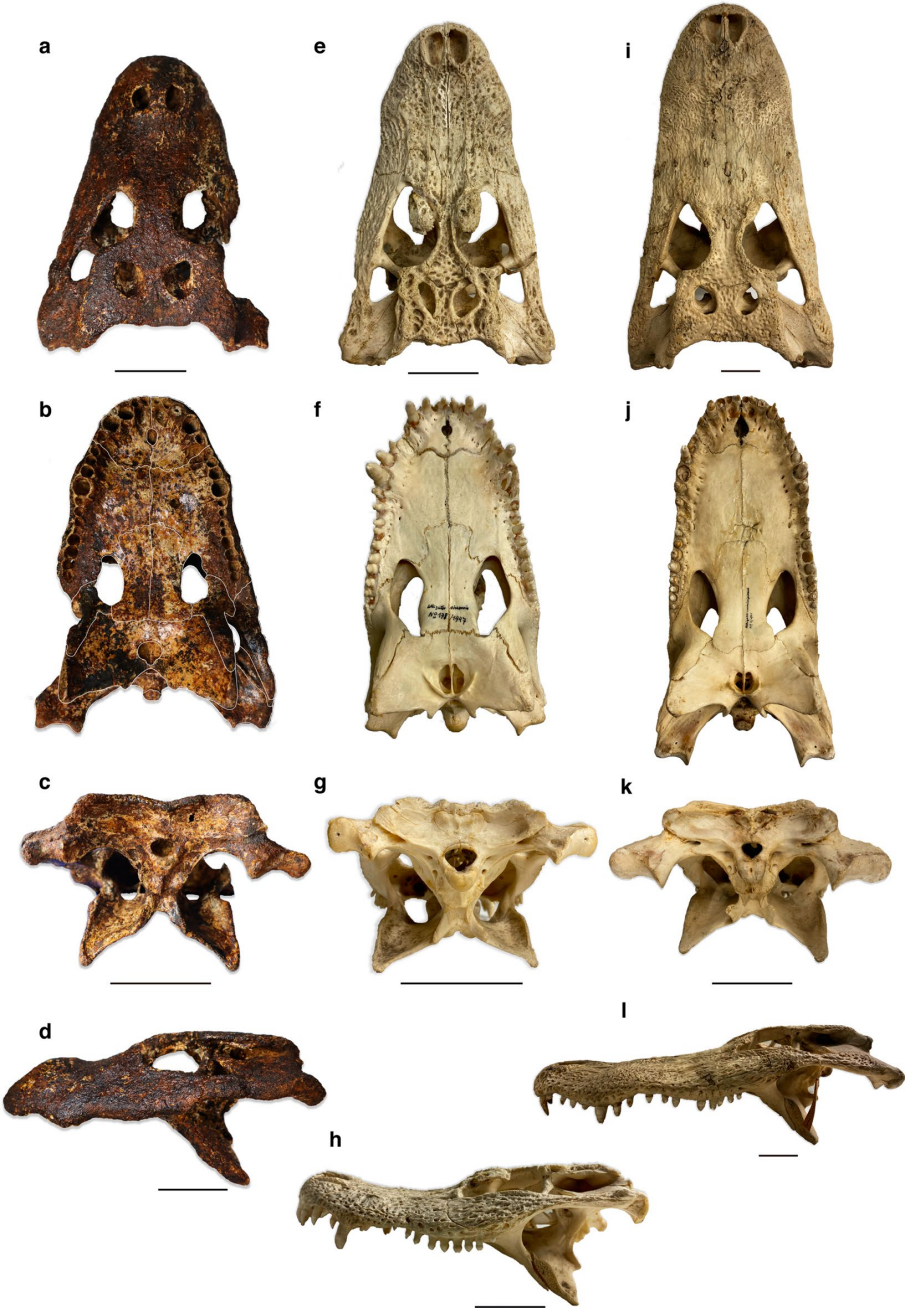
Comparison between the skulls of *Alligator munensis* sp. nov. holotype (DMR-BSL-2011-2) (**a**–**d**), *Alligator sinensis* (SNSB 178/1947) (**e**–**h**), and *Alligator mississippiensis* (SNSB 4/1921) (**i**–**l**) in dorsal, ventral, occipital and left lateral views from top to bottom, respectively. Scale bars: 5 cm.

**Figure 8:**
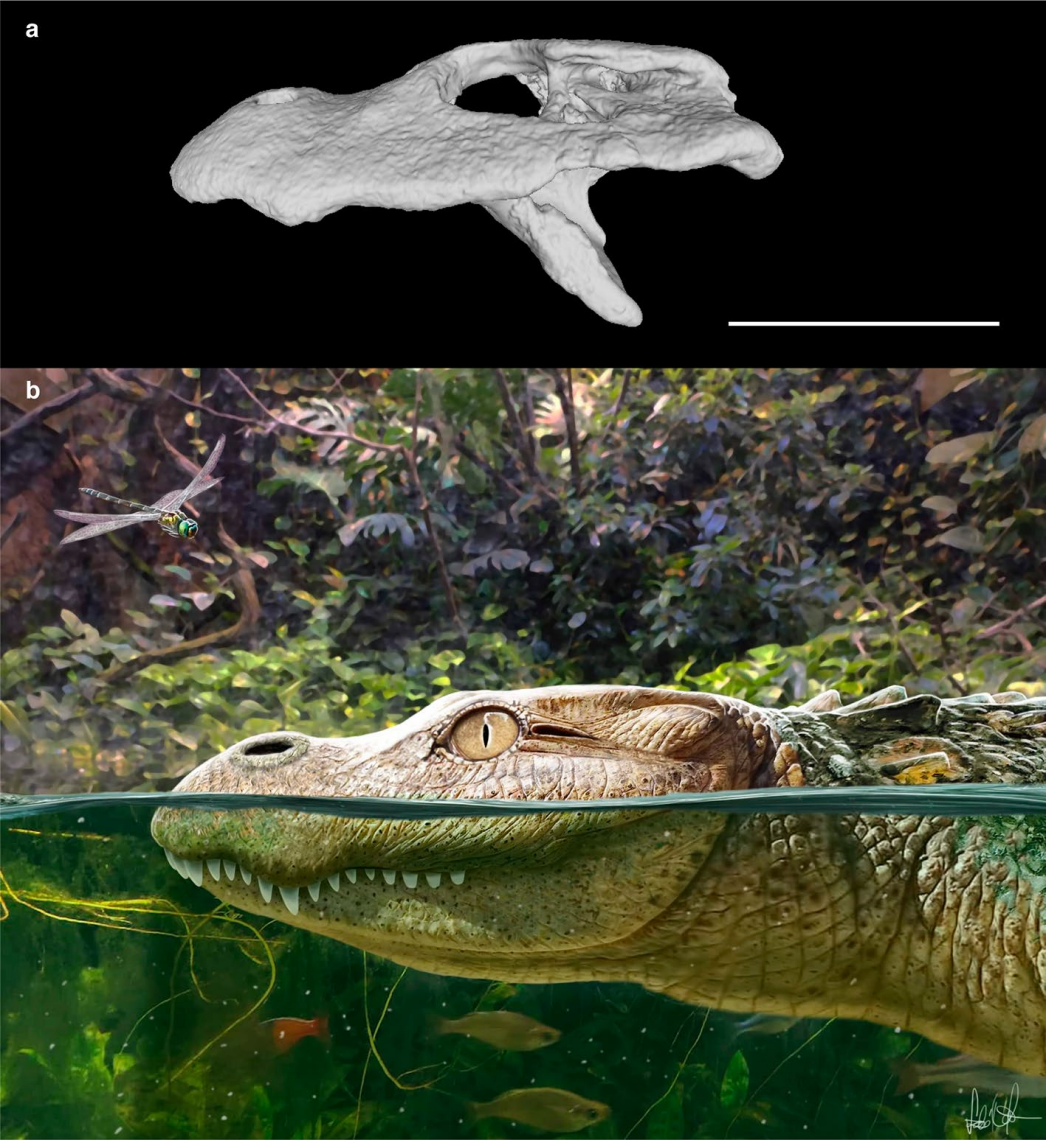
Virtual representation of *Alligator munensis* sp. nov. holotype (DMR-BSL-2011-2). (**a**) Digital reconstruction and (**b**) artistic reconstruction, both in left lateral views. Art by Márton Szabó. Scale bar: 10 cm.

*Alligator munensis* sp. nov. (Figs. 2, 3, 4, 5, 6, 7, 8)

*Alligator* cf. *sinensis* Fauvel^1^: Claude et al.^12^, page 126, plate 3.

### Etymology

The specific name *munensis* refers to the Mun River, close to the locality where the specimen was found in northeastern Thailand.

### Holotype

DMR-BSL-2011-2 comprises a nearly complete skull, missing only a few elements of the right side, such as the jugal and quadratojugal, and the dentition.

### Horizon and locality

Ban Si Liam locality, Non Sung district, Nakhon Ratchasima Province, Thailand, sandy layer 2 m below the surface (Fig. 1c).

### Diagnosis

*Alligator munensis* is diagnosed as an *Alligator* based on the external nares bisected by the nasals; *Alligator munensis* can be distinguished from all other extinct and extant *Alligator* species (i.e. *A. hailensis*, *A. mcgrewi*, *A. mefferdi*, *A. mississippiensis*, *A. olseni*, *A. prenasalis*, and *A. sinensis*) by the following combination of characters (autapomorphies marked with asterisk): anteroposteriorly compressed skull; significant posterior retraction of the external nares on the dorsal surface of the snout*; mediolaterally thick internarial bar resulting in small and circular external nares*; reduced maxillary dentition containing only 12 alveoli; dorsal surface of the nasals markedly concave on its anterior portion immediately posterior to the external nares*; presence of a smooth sagittal midline crest on the nasals*; presence of a sagittal midline crest on the posterior portion of frontal; frontal slightly convex and lacking upturned margins*; presence of a smooth midline crest on the parietal; presence of acute dorsal indentation on the parietal in occipital view; small and elliptical shaped incisive foramen; lateral process of the palatine not reaching the anterior margin of the suborbital fenestra; lateral palatine shelf forming a pointed tip; pterygoid excluded from the posterior margin of the suborbital fenestra by broad ectopterygoid-palatine suture*; absence of pterygoid “neck” around the posterior margin of the choana; bisected choana; posterior rims of the choana mediolaterally constricted; prominent quadrate condyles*.

### Comparative description

The following description will compare the cranial morphology of *Alligator munensis* to that of other extinct and extant species of *Alligator* (*A. mcgrewi*^28^; *A. luicus*; *A. mefferdi*^29^; *A. mississippiensis*; *A. olseni*^30^; *A. prenasalis*^31^; and *A. sinensis*). The holotype skull (DMR-BSL-2011-2) of *Alligator munensis* presents exceptional three-dimensionality, almost complete except for missing the dentition, the right jugal, the quadratojugal, and fragments of the braincase. The skull is triangular shaped in dorsal view, markedly compressed anteroposteriorly and particularly deep at the level of the external nares (altirostral skull sensu Salisbury and Willis^32^). The dorsal surface of the skull is ornamented, characterised by small rounded pits of varying size scattered along the cranial bones, gradually becoming less pronounced in the anterior portion of the snout. Dorsally, some of the sutures are difficult to trace owing to a thin iron-oxide layer covering large parts of the skull. However, we were able to reconstruct some of these using CT-scan imaging.

#### External naris

The external naris morphology of *Alligator munensis* is unique compared to other *Alligator* species (as well as to most crocodyliforms) in its anterior margin being retracted to the level of the occlusal pit on the premaxilla-maxilla suture (Figs. 2a,b, 3a,e, 4). In other species of *Alligator*, the anterior margin of the external naris is in line with the level of the third premaxillary tooth. The external naris of *A. munensis* is subcircular instead of the commonly teardrop-shaped outline seen in *Alligator* spp. and the apertures are separated from one another by an unusually wide internarial bar formed mainly by the nasals with significant contributions from the premaxillae. In other species of *Alligator*, the internarial bar is thin and has a short contribution from the premaxillae. The internarial bar of *A. munensis* forms a dorsoventrally high wall that is partially separating the narial cavity (Fig. 4c). Moreover, the posterior border of the external naris of *A. munensis* composed by the premaxillae and the nasals is raised, as in *A. sinensis* and *A. mcgrewi*.

#### Orbit

The orbits are more rounded compared to most other *Alligator* species except *A. prenasalis.* Upturned orbital margins of the frontal are absent. (Fig. 2a,b).

#### Supratemporal fenestra

The supratemporal fenestra is oval shaped resembling those of other *Alligator* species, except for *A. sinensis* which presents a more constricted fenestra. However, the supratemporal fossa (i.e. the region composed of unornamented bone surfaces immediately ventral to the supratemporal fenestrae, sensu Holliday et al.^33^) of *A. munensis* and *A. sinensis* are similar in being broadly exposed except for the anterior portion bearing the parietal and postorbital (Figs. 2a,b, 3d). The interfenestral bar is flat and broad as in *A. mississippiensis*, *A. prenasalis*, *A*. *mcgrewi*, and *A*. *olseni*, as opposed to the constricted and laterally upturned condition of *A. sinensis* and *A. mefferdi*.

#### Suborbital fenestra

The suborbital fenestra is elliptical and relatively short, reaching anteriorly to the level of the tenth maxillary alveolus (Figs. 2c,d, 3c). The suborbital fenestra is composed of the maxilla, palatine and ectopterygoid, with the maxilla forming the anterolateral to anteromedial border, without a contribution from the palatine. This condition is present in *A. mefferdi*, whereas in the remaining *Alligator* species the anterior border of the suborbital fenestra receives a lateral process of the palatine. The ectopterygoid completes the remaining lateral margin and participates at least for half of the posterior border of the fenestra. Additionally, the lateral margin of the suborbital fenestra exhibits a medial projection consisting of the maxilla and the ectopterygoid, a condition only present in *A. mcgrewi* and *A. sinensis* among *Alligator* species. The medial portion of the posterior border is formed by the palatine, excluding the pterygoid from the posterior border of the suborbital fenestra (Figs. 2c,d, 3c). In some specimens of *A. mississippiensis*, the anterior margin of the pterygoid has reduced participation in the fenestrae, in contrast to *A*. *sinensis*, where the pterygoid is involved.

#### Choana

The choana of *A. munensis* differs from the living *Alligator* species in two main aspects: it lacks the raised posterior margin and is semicircular in outline with a constricted posterior margin (Fig. 2c,d, 3f) instead of being elliptic. In *A. mcgrewi*, the constriction is present but less developed. The choanal septum is partially preserved in *A. munensis* and may be incomplete; whether the septum reaches ventrally the surface of the pterygoid cannot be assessed (Fig. 3f).

#### Premaxilla

The premaxilla is deep and prominent and is lacking a notch on the dorsal surface laterally to the external nares (Fig. 3a). In other *Alligator* species, the dorsal surface of the premaxilla bordering the lateral margin of the external naris presents a prominent notch (a groove), a character considered as an unambiguous synapomorphy for the genus^4^. The posterior premaxillary process extends approximately to the level of the third maxillary alveolus.

The external nares form only a third of the length of the premaxillae as opposed to other species of *Alligator* where they extend almost two thirds the length of the elements (Fig. 2a,b, 3a). Ventrally, the premaxilla presents five alveoli, with the third and fourth being the largest, whereas the remaining ones are markedly smaller. The fourth alveolus is the largest of the premaxillary teeth but only slightly larger than the third. Four occlusal pits are present medially to the premaxilla alveolar margin. The pit for the insertion of the first and fourth dentary teeth are the deepest and the diameter of the fourth is comparable to the alveoli of the fourth maxillary tooth (Fig. 2c,d, 3b). The incisive foramen is small and oval and shifted posteriorly from the anterior alveolar margin. Its anterior margin reaches the posterior border of the occlusal pit for the reception of the first dentary tooth and the posterior margin reaches the level of the fifth premaxillary alveolus (Fig. 3b). Furthermore, the suture between the premaxillae posterior to the incisive foramen is longer than the foramen (Fig. 3b). Among *Alligator* species, a small incisive foramen and long premaxillary suture posterior to it is otherwise present in *A. sinensis* whereas in *A. mefferdi*, *A. mcgrewi*, *A. mississippiensis*, and *A. prenasalis* the incisive foramen is reaching the level of the occlusal pit for the reception of the fourth dentary tooth. *A. olseni* has an intermediate condition.

#### Maxilla

The maxilla is dorsoventrally tall and presents a rostral ridge (sensu Rio & Mannion^34^) laterally extending from the anterior portion of the lacrimals to the level of the fourth maxillary tooth (Figs. 3e), an autapomorphic condition for *A.munensis* among *Alligator* species. Alongside the medial margin of the maxillary tooth row, shallow occlusal pits are present up to between the eighth and ninth maxillary alveoli (Fig. 2c,d). A maxillary process is present posterior to the last maxillary alveolus contacting the ectopterygoid and the jugal, as in *A. mississippiensis*, *A*. *mcgrewi*, *A. olseni* and *A. prenasalis*. Posteriorly, the palatine-maxilla suture extends from the seventh to the 10th maxillary alveoli as in *A. sinensis* and in *A. mcgrewi*, differing from the suture of *A. mefferdi*, *A. mississippiensis*, *A. olseni*, and *A. prenasalis* where it extends from the ninth to the 12th maxillary alveoli. The posterior margin of the maxilla forms the entire anterior and anterolateral border of the suborbital fenestra and projects medially inside the fenestra by a rugose process that also contacts the ectopterygoid. The rugose process of the maxilla is also present in *A. sinensis* and *A. mcgrewi*. The toothrow is reduced compared to other *Alligator* species; there are only 12 alveoli in the maxilla. The largest maxillary alveolus is the fourth, followed by the ninth to 11th, which are markedly enlarged in comparison to the remaining ones.

#### Nasal

The nasals are short and broad elements with strongly upturned anterior portions and making the area immediately posterior to the naris considerably depressed (positioned ventrally to the level of the internarial bar). The proportions are comparable to that of *A. sinensis*, *A. mcgrewi*, and basal short-snouted alligatorines (see Fig. 13 in Brochu^4^). The nasals are partially bisecting the premaxillae in the broad internarial (Fig. 4b,c). The nasal portions of the internarial bar form a deep vertical septum that bisects the nasal cavity almost up to half of its dorsoventral extension (Fig. 4c). Immediately posterior to the naris, the nasals and premaxilla form a raised border (Fig. 2a,b). This condition is also present in *A. sinensis* and *A. mcgrewi*. Among *Alligator* spp., a unique condition of *A. munensis* is a shallow sagittal crest present along the midline contact of the nasals (Fig. 3e).

#### Jugal

The jugal is only preserved on the left side but the concavo-convex suture with the maxilla is visible on the right side (Fig. 5 a,b). In lateral view, the jugal forms a linear ventral margin of the orbit and the transition of the infraorbital to the infratemporal portions of the jugal is marked by a pronounced step, another autapomorphy of this taxon. The infratemporal bar is relatively thick presenting a straight ventral outline (Fig. 5 c,d). A straight ventral margin of the infratemporal bar is also observed in *A. prenasalis*. The infratemporal bar comprises most of the ventral border of the infratemporal fenestra, except for the posteroventral corner formed by the quadratojugal (Fig. 5c,d).

#### Lacrimal

Both lacrimals are preserved, however the dorsal outline and suture with the prefrontal and maxilla are difficult to precisely trace. As a consequence of a deep skull, the lacrimals are more laterally positioned in comparison to other *Alligator* species. CT scan images reveal a lacrimal-maxilla contact positioned anteriorly to the jugal (Fig. 4d,e), but resolution is not sufficient to track the exact articulation among lacrimal, prefrontal, and maxilla and therefore the reconstructed lacrimal in Fig. 2b is speculative and merely follows the condition in most alligatorines^4^.

#### Prefrontal

The prefrontals are preserved on both sides, but similarly to the lacrimal, the sutures are difficult to be traced. The prefrontals bear a pair of smooth rostral ridges (sensu Rio and Mannion^34^) that together form a low spectacle close to the level of the anterior orbital margin. A low spectacle (sensu Rio and Mannion^34^) is also present in all *Alligator* species except *A. mcgrewi*. The spectacle in *A. munensis* is positioned slightly more anteriorly compared to other *Alligator* species where it is positioned posterior to the anterior orbital margin. Owing to the subcircular orbits, the margins of the prefrontal and frontal are more arched than in other *Alligator* species (Fig. 5). On the right lateral side, a partially preserved prefrontal pillar is present, being posteriorly slightly convex at its dorsalmost portion.

#### Frontal

The frontal presents a uniquely broad and arched interorbital bar lacking upturned lateral margins. Upturned orbital margins are also absent in *A. prenasalis*. The dorsal surface of the frontal of *A. munensis* bears a thin midline crest posteriorly (Figs. 2a,b, 3d, 5), which is an autapomorphy of this species. The frontoparietal suture extends anterior to the supratemporal fenestra (Fig. 4a). The exact limits of the anterior process of the frontal cannot be fully assessed.

#### Postorbital

The postorbital is wide and because of the iron oxide encrusting, it is unknown if a sulcus between the medial and lateral margins were present dorsally (Fig. 2a,b, 3d). In *A. sinensis*, the lateral margin develops a shallow crest whereas the medial margin is less pronounced but also upturned; together these delimit a sulcus. In *A. munensis*, a pronounced crest is absent but we cannot rule out the presence of a sulcus. The dermal part of the postorbital overhangs the supratemporal fenestra thereby obscuring the anterior margin of the supratemporal fossa. This is a condition shared with *A. sinensis*. The postorbital contacts the parietal medially along the anterior margin of the supratemporal fenestra, excluding the frontal from contacting the fenestra. The postorbital bar is inset from the dorsolateral margin of the jugal.

#### Squamosal

The squamosals are flat and prominent elements of the broad skull table unlike *A. sinensis*, where the lateral and posterior margins are decorated by a pair of shallow crests, the margins are smooth in *A. munensis* (Fig. 2a,b, 3d). The squamosal prongs at the dorsal contact with the quadrate are mostly covered by the lateral margin of the skull table (Fig. 2a,b) as in *A. mcgrewi*, differing from the dorsally exposed prongs of other *Alligator* species (Fig. 7a,d,g). Laterally, the squamosal composes the posterior border of the external auditory meatus.

#### Parietal

The dorsal surface of the parietal has a smooth midline crest (Fig. 3d), a condition also present in *A. sinensis* and *A. mcgrewi*. The posterior border of the parietal reaches the limit of the skull table excluding the dorsal exposure of the supraoccipital. The supratemporal fossa is broadly exposed along the medial margins of the parietal except at the parietal-postorbital suture, as also observed in *A. mcgrewi* and in some specimens of *A. sinensis* (IRScNB 13,904; SNSB 178/1947; Fig. 7d). The parietal has a sagittal midline depression as in *A. mississippiensis* and *A. mefferdi*. An opposite condition (i.e. flat dorsal outline of the parietal in occipital view) is seen in *A. sinensis*, and variable in *A. mcgrewi*. In the remaining fossil *Alligator* species, the parietal morphology is affected by poor preservation.

#### Quadratojugal

Only the left quadratojugal is preserved. It forms the posteroventral border and the posterior margin of the infratemporal fenestra (Fig. 5c,d) and is similar to other *Alligator* species in tapering dorsally.

#### Quadrate

The quadrate is preserved on both sides and is characterised by a markedly concave intercondylar area with strong, ventrally directed hemicondyles, clearly visible in dorsal and ventral views (Fig. 2). In other species of *Alligator*, the area between the lateral and medial hemicondyles are less concave (Fig. 7d,e,g,h). The shape and size of the quadrate condyles are similar to other *Alligator* species, with the lateral condyle slightly larger than the medial, except for *A. sinensis*. The lateral condyle of *A*. *sinensis* has a unique morphology in having the lateral condyle dorsoventrally twice the size of the medial condyle. In occipital view, the portion of the quadrate bordering the braincase is not visible. This is like in all other species of *Alligator* except for *A. sinensis*. The foramen äerum is positioned on the dorsal surface of the quadrate ramus (Fig. 6b), as in all other alligatoroids^4^. The following crests for the insertion of jaw muscles^35^ are preserved at the ventral surface of the quadrate: the crest A, positioned along the quadrate-quadratojugal suture, is well-developed and prominent as in *A. mcgrewi*, *A. prenasalis* and *A. sinensis*, differing from a smooth crest of *A. mississippiensis*, *A. mefferdi* and *A. olseni*. The crest B of *A. munensis* extends posteriorly on the quadrate ramus being continuous with the crest B' as in *A. mcgrewi* and *A. sinensis*, although in *A. sinensis* the crest B is well-developed and convex, a condition not present in *A. munensis* or in any other *Alligator* species. Finally, the quadrate ramus is exposed beneath the quadratojugal (Fig. 5c,d).

#### Palatine

The palatines are wide and compose the medial and posteromedial margins of the suborbital fenestra. The anterior process of the palatine (i.e. palatine-maxilla suture) reaches the level of the seventh maxillary tooth (Figs. 2 c,d, 3c) as in *A. sinensis* and *A. mcgrewi*. The anterior process of the palatine is quadrangular in shape as seen in other *Alligator* species. The anterolateral process of the palatine is reduced compared to all other *Alligator* species and does not reach the anterior margin of the suborbital fenestra (Fig. 3c). Posteriorly, the palatine shelf strongly projects laterally forming almost an angle of 90 degrees with the sagittal plane of the palatine. A less pronounced lateral projection of the palatine shelf is also observed in *A. mcgrewi* (AMNH 7905). The palatine comprises the posteromedial border of the suborbital fenestra and contacts broadly the ectopterygoid, which completes the posterior border of the fenestra, excluding the pterygoid (Fig. 3c). The contact of the palatine and ectopterygoid hinders the participation of the pterygoid in the margin of the suborbital fenestra, another unique condition of *A. munensis*. In *A. mississippiensis* the palatine and ectopterygoid are broadly separated by the pterygoid (SZ 1057), but in one examined specimen (SNSB 4/1921) the ectopterygoid approaches the palatine, being briefly separated by the anterior margin of the pterygoid (Fig. 7h). Finally, the palatine-pterygoid suture is located far posterior to the suborbital fenestra (Fig. 3c), like in *A. mississippiensis* and *A. mcgrewi*.

#### Ectopterygoid

As in all alligatoroids, the ectopterygoid is not participating in the maxillary medial alveolar margin. It is partially composing the lateral and posterior margins of the suborbital fenestra (Figs. 2 c,d, 3c). The anterior portion of the ectopterygoid extends to the level of the 11th alveolus. The ectopterygoid broadly contacts the palatine behind the suborbital fenestra, an autapomorphic condition for *A. munensis*. The posterior end of the ectopterygoid wing does not reach the posterior end of the pterygoid wing. Additionally, the ectopterygoid terminates at the base of the postorbital bar, as commonly observed in *Alligator*.

#### Pterygoid

The pterygoids are overall similar to other *Alligator* species except for being excluded from the posterior border of the suborbital fenestra (Fig. 2 c,d). The pterygoid contacts anteriorly the palatine and anterolaterally the ectopterygoid. The choana lacks a ‘neck’, presenting its posterior margin markedly constricted, and there is a pair of shallow depressions around the choanal aperture (Fig. 3f). A constricted posterior margin of the choana is also seen in *A. mcgrewi*, although not as marked as in *A. munensis*. In occipital view, the posterior process of the pterygoid is short and projects ventrally as in *A. sinensis* and *A. mcgrewi*. Dorsally to the process, the pterygoid articulates with the parabasisphenoid.

#### Parabasisphenoid

As in other species of *Alligator*, the parabasisphenoid is a thin, subtriangular element located between the basioccipital and the pterygoid and forming the anterior wall of the medial and lateral eustachian foramen. The parabasisphenoid is not exposed in the lateral braincase wall. Ventrally, it extends along the posterior surface of the pterygoid as exposed in occipital view (Fig. 6). The posterior portion of a partially preserved parabasisphenoid rostrum can be observed in right lateral view (Fig. 5a,b).

#### Basioccipital

The basioccipital is morphologically similar to other *Alligator* species, in which the basioccipital tubera is wide, presenting a pronounced midline crest almost reaching the ventral portion of the basioccipital condyle (Fig. 6). The occipital condyle of *A. munensis* is relatively small and more spherical compared to other *Alligator* species.

#### Supraoccipital

The supraoccipital is subtriangular shaped, excluded from the dorsal surface of the skull table, and covered dorsally by the parietal (Fig. 6). A pair of shallow depressions are present laterally on the occipital surface. The lateral portion of the supraoccipital is lacking any protuberances and the dorsal articulation with the parietal and squamosal forms a continuous surface, preventing the exposure of the postemporal fenestra (sensu Kuzmin et al.^36^), an autapomorphic condition for *A. munensis*. The midline of the occipital surface is slightly posteriorly pronounced, but not forming a marked midline crest, distinguishing *A. munensis* from other *Alligator* species.

#### Exoccipital/paroccipital process

The exoccipital composes the lateral and dorsal margins of the foramen magnum and extends slightly ventral to the basioccipital condyle, but not reaching ventrally the basioccipital tubera (Fig. 6), as commonly observed in *Alligator*. Two pairs of foramina, the one for the cerebral carotid artery lateral to the basioccipital condyle and one for the passage of cranial nerves IX–XI are positioned more dorsally at the level of the ventral margin of the foramen magnum^35,36^. The paroccipital process is laterally projected as in most *Alligator* species, differing from a marked dorsolaterally oriented process of *A. sinensis*. The dorsal margin of the foramen magnum at the contact of the exoccipitals is marked by a small protuberance, also commonly observed among *Alligator* species, except for *A. sinensis.*

#### Braincase

Some elements of the braincase are preserved but incomplete. The foramen ovale is present and is composed anteriorly by the laterosphenoid, ventrally by the pterygoid and posteriorly by the quadrate (Fig. 5a,b). The preservation around the foramen hampers the precise observation of the prootic exposure. Only the posterior portion of the laterosphenoid (e.g. lateral bridge, sensu Holliday and Witmer^37^) is preserved.

## Discussion

### Taxonomy of *Alligator munensis*

A comprehensive phylogenetic analysis of DMR-BSL-2011-2, the holotype of *Alligator munensis*, including an increased taxon sampling of *Alligator* spp., is in preparation and will be published elsewhere. The morphology of the skull nevertheless clearly implies an *Alligator* closely related to extant *A. sinensis*. Alligatoroid synapomorphies include a laterally shifted foramen äerum, a maxillary shelf separating the posterior toothrow from the ectopterygoid, whereas the full premaxillary-maxillary overbite, the largest 4th maxillary tooth, and a fronto-parietal suture entirely on the skull table diagnose the morphological features of Alligatoridae. Among alligatorids, only *Alligator* has bisected external nares^4,38^ as also present in *A. munensis*. *Alligator munensis* shares several apomorphic characters with *A. sinensis* to the exclusion of *A. mississippiensis*: (*i*) small incisive foramen occupying one third of the length of the premaxilla (Figs. 3b, 6a,b); (*ii*) ridge on the dorsal surface of the parietal (Fig. 3d); (*iii*) the presence of a raised posterior margin of the external nares (Fig. 3e); (*iv*) rugose ventral surface of lateral maxillary shelf projecting into the suborbital fenestra (Fig. 3c); (*v*) small, tubera-like posteroventrally projecting posterior pterygoid processes. These character states are furthermore shared with *A. mcgrewi* from the Miocene of North America (except for the small incisive foramen and raised posterior margin of the external nares), in addition to the shelf of the palatine projecting laterally at the posterior border of the suborbital fenestra (Fig. 3c), a condition shared exclusively between *A. munensis* and *A. mcgrewi*. In *A. luicus* from the Miocene of China, characters (*ii*) and (*iii*) are present but the rest of the character states are not preserved in the only known specimen.

DMR-BSL-2011-2 was preliminary reported by Claude et al.^12^ who tentatively referred it to *Alligator* cf. *sinensis* based on the presence of a straight posterior skull margin; long distance between the posterior margin of the skull table and the temporal fenestra; broad (wider than long) skull table; and a well-developed internarial bar. Subsequent preparation (not possible at the time) and detailed description in the present study reveal that a highly distinct morphology warrants a new species. *A. munensis* differs from *A. sinensis* in having a slightly convex dorsal surface of the frontal lacking upturned margins; reduced dentition with 12 instead of 14 maxillary alveoli; lacking a convex crest B of the quadrate; lacking a crest with a ventral protuberance on the ventral margin of the exoccipital dorsal to the cranioquadrate passage; lacking a pair of horns formed by the parietal and squamosal on the posterodorsal margin of the skull table (potentially correlated to the latter character); lacking a ridge above the dorsal rim of the lateral groove of the squamosal; and lacking a crest on the dorsal surface of the lateral margin of the palatine (Fig. 7). Additionally, *Alligator munensis* can be distinguished from all other fossil and extant *Alligator* species by the presence of the following autapomorphic characters: posteriorly retracted circular and reduced external nares; a wide internarial bar; a sagittal crest along the midline contact of the nasals; frontal slightly convex and lacking upturned margins; pterygoid excluded from the posterior margin of the suborbital fenestra by a broad ectopterygoid-palatine suture; and prominent quadrate condyles. These autapomorphies imply that *Alligator munensis* was not ancestral to *Alligator sinensis* and rather represent a divergent, possibly allopatric species.

### Biogeographical implications

As discussed by Claude et al.^12^, the unexpected presence of an alligatorid in northeastern Thailand requires explanation. The tentative referral of DMR-BSL-2011-2 to *A.* cf *sinensis* posed a biogeographic enigma because the geographically closest historical occurrences of *A. sinensis* come from the Yangtze and Xi river systems^39,40^ that only approach the Mekong and Chao Phraya systems of Thailand along their upper sections at high elevations, unfavourable for alligators^12^. Nevertheless, as we here demonstrate, the specimen DMR-BSL-2011-2 confidently represents a separate species albeit with close affinities to *A*. *sinensis*. The highly distinct morphology of *A. munensis*, on the other hand, is more consistent with relatively deep divergence from *A*. *sinensis*. If that is the case, the presence of *Alligator* in Thailand may be explained by a hypothetical ancestor distributed in the lowlands of both the proto Yangtze-Xi and Mekong-Chao Phraya river systems that was subsequently split into separate, vicariant species due to the accelerated Miocene uplift of the eastern Tibetan Plateau^41^, fully hindering dispersal between these drainage basins. The Mun river, the locality of the *A. munensis* specimen described herein, feeds the Mekong today but was connected to the proto-Chao Phraya river in the past^41–43^. A time-scaled phylogenetic analysis in preparation by GD and MR will be a useful way to test this scenario.

### Remarks on the retracted external nares of *Alligator munensis*

Unlike other crocodylians, *A. munensis* bears unique small, rounded external nares that are posterodorsally retracted and are bisected by a wide internarial bar (Figs. 2a,b, 8). In Crocodylia, *Purussaurus* spp., possess retracted external nares that are further characterised by the posterior expansion of the posterior margin^44^, a condition absent in *A. munensis*. Though far not to the extreme seen in *A. munensis* and with large narial apertures retained, some relatively broad-snouted species of *Crocodylus* (e.g., *C. palustris* and *C. thorbjarnarsoni*^45^) furthermore possess slightly retracted nares. Admittedly, our survey was not extensive and slightly retracted nares may be present in other taxa as well. Pronounced retraction of the external nares is often associated with an adaption of the nasal system that facilitates respiration in aquatic environments as documented in the evolution of cetaceans^46^ and commonly observed in marine reptiles such as ichthyosaurs, plesiosaurs, mesosaurs, and metriorhynchid crocodylomorphs^47,48^. However, it is highly unlikely that *A. munensis* was marine given the depositional environment and geographic origin of the fossil specimen. Moreover, *Alligator* species lack lingual salt-excreting glands^49,50^ and cannot survive indefinitely in hyperosmotic conditions^51^. Natural habitats of the Chinese alligator were low-elevation areas, alluvial floodplains with a variety of wetlands including marshes, ponds, and streams, although these have been significantly altered by human activities^39^. How *A. munensis* would have taken advantage of its unique external narial morphology, if it were living in similar habitats (consistent with depositional environments), remains unclear.

### Comments on the evolution of dietary specialisation in alligatorines

While the dentition of *Alligator munensis* is unknown, enlarged alveoli 9–11 and the deep, blunt snout may provide some clues to feeding ecology (Fig. 3c). In several short-snouted alligatoroids, the posterior maxillary/dentary alveoli are considerably enlarged and contain more globular^52,53^ or blunt and labiolingually compressed teeth^54^ relative to more anterior alveoli posterior to the 4th maxillary/dentary alveolus. The presence of similar alveolar morphology thus implies that *A. munensis* possessed enlarged posterior teeth of either of these morphotypes (Supplementary Fig. 4). The presence of a robust, deep skull, wide posterior process of the maxilla lateral to the suborbital fenestra, robust ectopterygoids, wide palatinal bridge between the suborbital fenestrae, and large and extended pterygoid flanges of *A. munensis* further suggest extensive origin surfaces for well-developed jaw adductor muscles and strong bite force consistent with a crushing type of dentition^55^. Although appearing in various lineages in Crocodylia including alligatoroids and crocodyloids^55^, a crushing dentition is plesiomorphic for alligatorines (total-group of *Alligator*), and has been used to exemplify a case for “specialised” taxa giving rise to “generalist” taxa (*Alligator* spp.) and thus breaking the "Law of the Unspecialised'' of Cope^56–58^. Cope's concept proposes that "specialised" features would not revert to a generalised condition and generalists could not evolve from specialists. Translating morphology to ecology is usually not straightforward for fossils, however (as also noted by Brochu^52^) “specialisation” may simply represent deviation from the ancestral anatomy and may not be necessarily associated with a distinct (narrower) niche. Likewise, in case of alligatorines, crushing dentition does not automatically imply more specialised ecology—in the absence of evidence to the contrary, it might as well be interpreted as adaptation for a more opportunistic diet with potential seasonal preferences of hard-shelled preys (broader niche). The macrocephalic turtle, *Platysternon megacephalum* may serve as an example: this species potentially takes advantage of its large head when seasonally consuming molluscs but it is otherwise an omnivorous species^59^. Moreover, crocodylians without enlarged (albeit stocky) posterior dentition occasionally prefer hard-shelled prey resulting in advanced dental wear, as has been reported for *Caiman latirostris*^60^. Information on the diet of *Alligator sinensis* is scarce; a single study reported snails as dominant prey^61^. *A*. *sinensis* does not have enlarged posterior teeth or alveoli but the crowns are slightly rounded, more so than in e.g. *Alligator mississippiensis*. Although there is no evidence of molluscs in the type locality of *A. munensis*, gastropod shell remains were reported from the late Middle Pleistocene site of Khok Sung^14^, a site which might have been close to Ban Si Liam both geographically and chronologically^18^ (Fig. 1).

Regardless of ecological function, the pattern of morphological simplification of the dentition in crown-group *Alligator* and the apomorphic development of a longer snout in the *A. mississippiensis* lineage^58^ is apparent. *A. munensis*, on the other hand, may represent an outlier by reverting to the ancestral condition of enlarged posterior crushing dentition characterising early “specialized” alligatorines. Intriguingly, crocodylians with enlarged globular/flattened dentition were common in the past and evolved independently in multiple lineages (e.g.^4,52,53,55,62^) but this particular morphotype is absent in the living fauna. *A. munensis* may have been one of the last examples of the crushing-dentition morphotype.

## Methods

### Comparative material

The comparative analysis of the present study was conducted using the following specimens: *Alligator mcgrewi* AMNH 7905, FMNH P26242; *Alligator mefferdi* AMNH 7016; *Alligator mississippiensis* SNSB 4/1921*, 2530/0*; SZ 1057*; *Alligator olseni* MCZ 1887, 1899; *Alligator prenasalis* YPM–PU 13799, MCZ 1014, 1015; *Alligator sinensis* AMNH 23899, 23901, 139673, 140775, IRScNB 13904; R23898; SNSB 178/1947*; and *Caiman crocodilus* SZ 10276*; in addition to published data (Brochu, 1999). Specimen numbers indicated with an asterisk were first hand studied, whereas the remaining specimens were studied through photographs.

### Digitalisation and imaging

Photographs were taken by G.D and M.R**.** CT scan image stacks were acquired using CT scanner Philips IQon Spectral CT (Advanced Diagnostic Imaging Center (AIMC), Mahidol University in Thailand), with voltage of 120 kV and current of 562 µA. A total of 1272 slices with thickness of 0.80 mm were generated and voxel size of 0.234375 × 0.234375 × 0.4 mm. Digital reconstructions and CT scan image stacks were processed using Amira software 3D 2021.1 (https://www.thermofisher.com/order/catalog/product/AMIRA) in the 2D/3D imaging/digitisation lab of the Centre of Visualisation, Digitalisation and Replication at the Eberhard Karls Universität Tübingen, Germany. Additional photographs of 3D models were made using the software MeshLab 2021.07 (https://www.meshlab.net/). Images were further processed in Adobe Photoshop CC (https://www.adobe.com/products/photoshop.html) and all drawings and figures were produced using Adobe Illustrator CC (https://www.adobe.com/products/illustrator.html).

### Nomenclatural acts

This work and the nomenclatural acts it contains have been registered in the proposed online registration system (ZooBank) for the International Code of Zoological Nomenclature. The ZooBank Life Science Identifier can be resolved and the associated information viewed through any standard web browser by appending the LSID to the prefix http://zoobank.org/. The LSID for this publication is: [urn:lsid:zoobank.org:pub:844D9DB3-98B2-40B6-9AE9-666B6B9C8DE5].

## Supplementary Information

Supplementary Information.

## Data Availability

All data generated or analysed during this study are included in this published article (and its Supplementary Information files). Raw CT scan data (DICOM stack format) is available on the following link to MorphoSource Ark repository: http://n2t.net/ark:/87602/m4/490374.
