# Supplementary material for: An extinct deep-snouted Alligator species from the Quaternary of Thailand and comments on the evolution of crushing dentition in alligatorids
Source: Sci Rep. 2023 Jul 13;13:10406. doi: 10.1038/s41598-023-36559-6 (PMC10344928; doi:10.1038/s41598-023-36559-6)
Supplement: Supplementary file 1 — Supplementary Information. [file 41598_2023_36559_MOESM1_ESM.pdf]

An extinct deep-snouted *Alligator* species from the Quaternary of Thailand and comments on the evolution of crushing dentition in alligatorids

Gustavo Darlim, Kantapon Suraprasit, Yaowalak Chaimanee, Pannipa Tian, Chotima Yamee, Mana Rugbumrung, Adulwit Kaweera, Márton Rabi

Supplementary Information

Content

1. Geological setting, faunal assemblage, and age……………………………………1

2. Supplementary figure for Discussion: maxillary alveolar morphology …….……….7

3. References……………………………………………………………………….8

Institutional abbreviations. AMNH, American Museum of Natural History, New York, New York, USA; DMR–Department of Mineral Resources, Bangkok, Thailand; YPM-PU, Yale Peabody Museum-Princeton University collection, New Haven, Connecticut, USA.

1. Geological setting, faunal assemblage, and age

The fossil site is located at Ban Si Liam, Mai Subdistrict, Non Sung district, Nakhon Ratchasima Province in northeastern Thailand (Fig. 1a, b). In 2005, the square-shaped pond with an area of 8 m long x 8.4 m wide x 2 m deep was dug out by the villagers and yielded some vertebrate fossils (Supplementary Fig. 1). Regarding the stratigraphic profile of Ban Si Liam (Fig. 1c), the dark-colored topsoil is 30 cm in thickness and organic-rich in content, underlain by yellowish medium- to fine-grained sands with the thickness of 2 m. Some fragments of ancient pottery and ceramics were collected from the topsoil but vertebrate fossils (nine specimens) were entirely found from the yellowish sandy layer that overlies a thin layer of indurated iron oxide (10 cm thick), followed by the yellowish clay at the lowermost part of the pond. Three reptile fossils included a fragment of a turtle carapace (DMR-BSL2011-1) and a nearly complete cranium of an alligator (DMR-BSL2011-2), both of which have been previously reported by Claude et al. (2011), as well as a crocodylian vertebra represented by a well-preserved

centrum and neural arch tentatively assigned as the fourteenth dorsal vertebra (DMR-BSL2011-3; Supplementary Fig. 2).

In addition to the alligator's skull described here in this study, fossils of two mammalian species collected from the same layer were identified as belonging to a wild water buffalo (*Bubalus arnee*) and a sambar deer (*Rusa unicolor*) based on the comparisons of morphological features and dimensions with extant comparative specimens and fossils recovered nearby (i.e. the late Middle Pleistocene fauna from Khok Sung, Suraprasit et al., 2016). Six mammalian remains included two mandibular specimens (DMR-BSL2011-4 and DMR-BSL2011-8), a horn core fragment (DMR-BSL2011-5), and a cervical vertebra (DMR-BSL2011-9) of a wild water buffalo *Bubalus arnee* as well as a mandible (DMR-BSL2011-6) and a femur (DMR-BSL2011-7) of a sambar deer *Rusa unicolor* (Supplementary Fig. 3).

Although the right mandible DMR-BSL2011-4 (designated as *Bubalus arnee*) possesses a very worn p3 to m2, the p3 shows a shallower posterior valley than a medial one, which is a typical feature of *Bubalus* (Suraprasit et al., 2021) (Supplementary Fig. 3a, b). The horn core fragment DMR-BSL2011-5 is suboval in cross-section outline with a more flattened surface on the anterior side (Supplementary Fig. 3c). The left mandible DMR-BSL2011-6, assigned to *Rusa unicolor*, is characterized by well-developed conids and stylids on cheek teeth as well as basal pillars on molars, similar to extant sambar deer (Suraprasit et al., 2016, 2021) (Supplementary Fig. 3d, e) The sizes of cheek teeth embedded in these two jaws (DMR-BSL2011-4 and DMR-BSL2011-6) are comparable to those of extant wild water buffalo and sambar deer specimens, respectively (Table.1) (see Suraprasit et al. (2016; figs 22 and 27) and Suraprasit et al. (2021; tab. 5) for more detailed measurements and comparisons). The right femur DMR-BSL2011-7 is similar in morphology and size to extant *Rusa unicolor* (see Suraprasit et al., 2021; appendix 10)

(Supplementary Fig. 3f). The fragmentary mandible DMR-BSL2011-8 preserves portions of a condyle, ascending ramus, and mandibular foramen and angle (Supplementary Fig. 3g). According to the size and shape, this mandibular fragment and the sixth cervical vertebra DMR-BSL2011-9 are assigned to *Bubalus arnee* (Supplementary Fig. 3h).

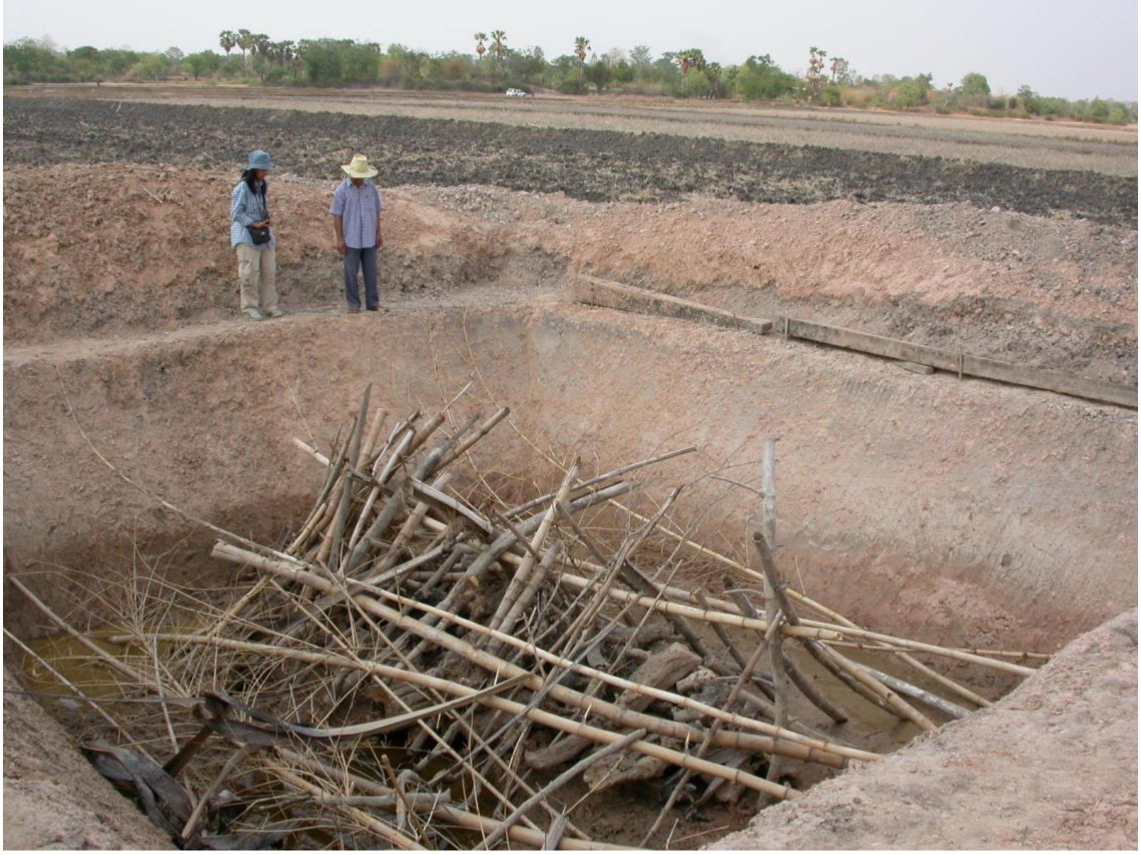

Supplementary figure 1. The fossil site of Ban Si Liam in Non Sung district (Nakhon Ratchasima), photo taken during the excavation.

Despite the ages of the possible Late Miocene to Pleistocene having previously been proposed by Claude et al. (2011), the two living mammal species contemporaneous with an alligator suggest a more limited range of faunal ages possibly spanning from the late Middle Pleistocene to Holocene because the presence of these taxa is congruent with fossils records from some late Middle Pleistocene localities in Thailand (Tham Wiman

Nakin (dated to >169 ka, Esposito et al., 1998, 2002; Suraprasit et al., 2021)) and Khok Sung (dated to either 217 or 130 ka, Suraprasit et al., 2016; Duval et al., 2019). Moreover, the stratigraphic position of a fossiliferous layer at Ban Si Liam is quite shallow (around 2 m below the surface, Fig. 1c), compared to other Late Miocene sedimentary deposits along the Mun River systems (i.e. around 10 to 20 m deep in Tha Chang sandpits, Chaimanee et al. (2004)).

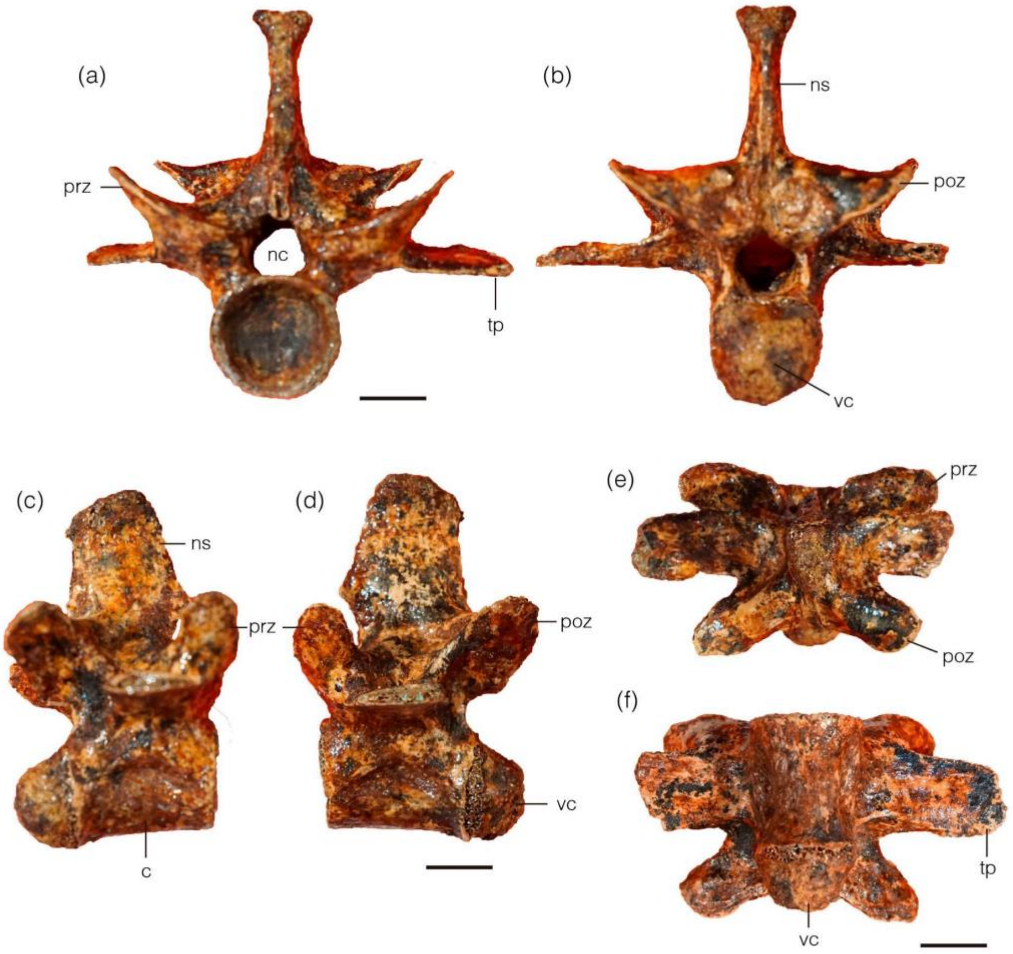

Supplementary figure 2. Crocodilian vertebra (DMR-BSL2011-3) tentatively assigned as the fourteenth dorsal vertebra in anterior (**a**), posterior (**b**), right lateral (**c**), left lateral (**d**), dorsal (**e**), and ventral (**f**) views. Abbreviations: c, centrum; nc, neural canal; ns, neural spine; poz, postzygapophysis; prz, prezygapophysis; tp, transverse process; vc, vertebral condyle. Scale bar: 1 cm. Figure generated using Adobe Illustrator CC.

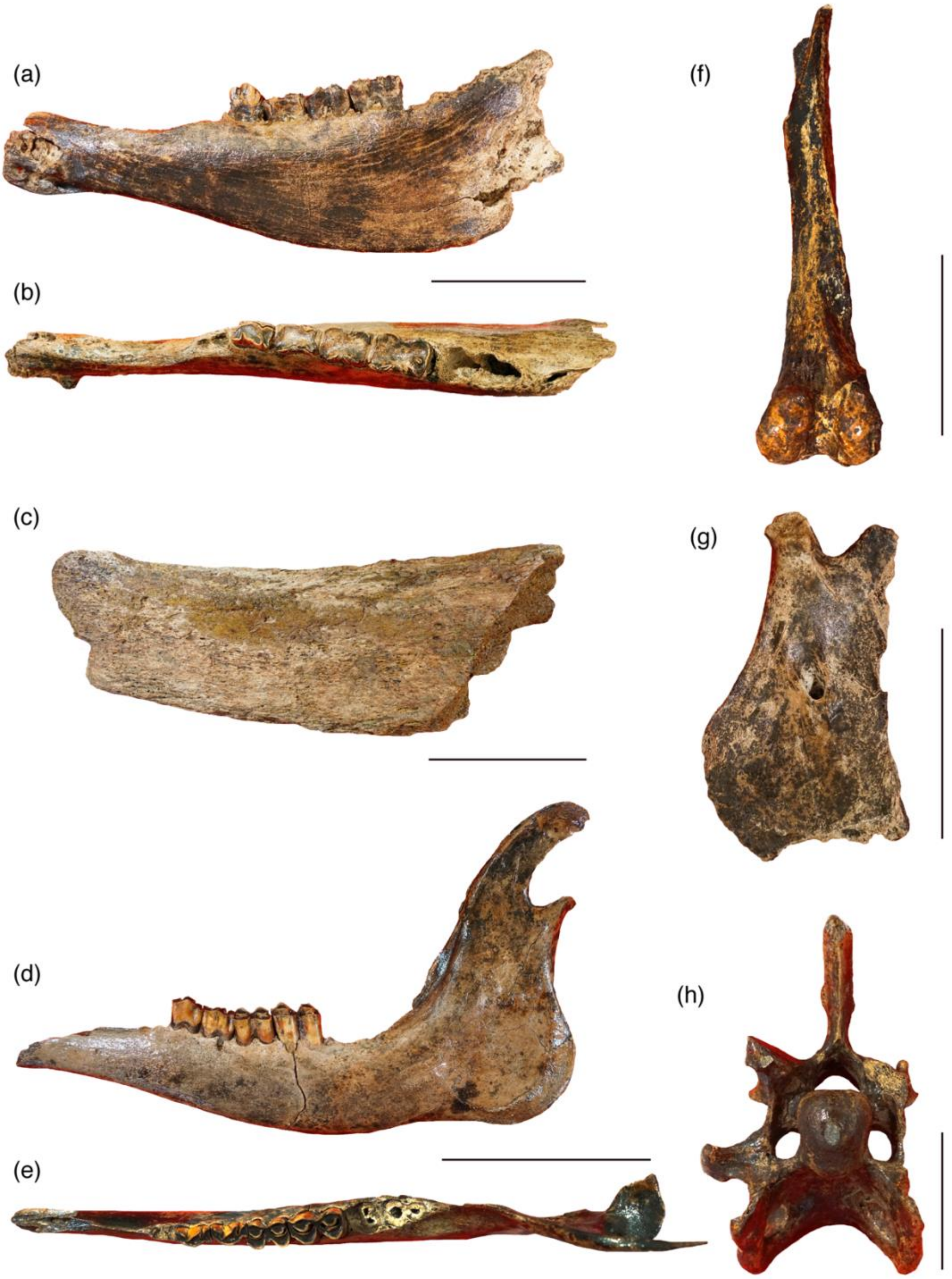

Supplementary figure 3. Mammalian remains from the fossil site of Ban Si Liam, same layer of *Alligator munensis* sp. nov., here assigned as *Bubalus arnee* (**a–c, g, h**), and *Rusa unicolor* (**d–f**). Right mandible (DMR-BSL2011-4) in medial (**a**) and occlusal (**b**) views; horn core fragment (DMR-BSL2011-5) in dorsal view (**c**); left mandible (DMR-BSL2011-6) in lateral

(**d**) and occlusal (**e**) views; femur (DMR-BSL2011-7) in posterior view (**f**); fragmentary mandible (DMR-BSL2011-8) in medial view (**g**); and cervical vertebra (DMR-BSL2011-9) in anterior view (**h**). Scale bar: 10 cm (a–g); 5 cm (h). Figure generated using Adobe Illustrator CC.

Table 1. Dental measurements of cheek teeth of ruminants from Ban Si Liam.

**Specimen no.**	**Teeth**	**L**	**W**
		**(mm)**	**(mm)**
Bubalus arnee			
DMR-BSL2011-4	p3 (right)	21.36	14.50
	p4 (right)	25.41	16.42
	m1 (right)	26.21	18.82
	m2 (right)	31.33	21.36
Rusa unicolor			
DMR-BSL2011-6	p3 (left)	14.80	8.62
	p4 (left)	15.22	9.66
	m1 (left)	18.80	12.55
	m2 (left)	22.92	14.43

2. Supplementary data for Discussion: alveolar morphology

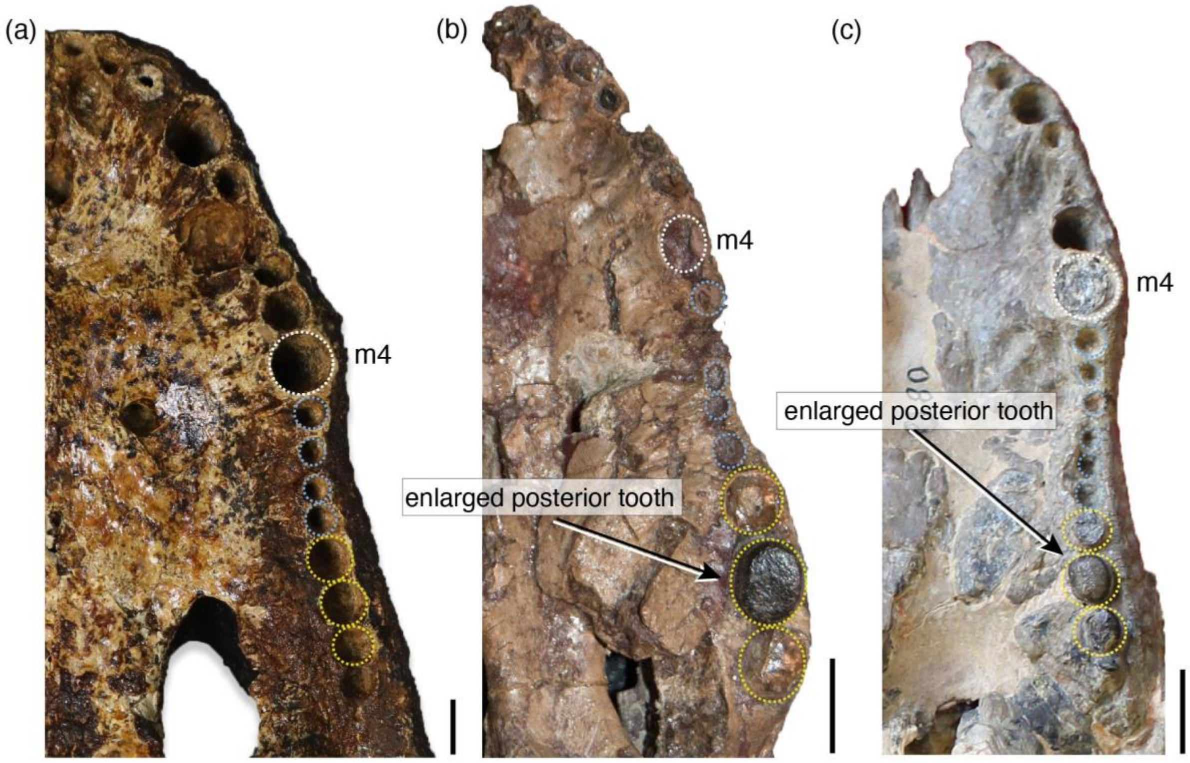

Supplementary figure 4. Ventral view of the palate showing maxillary alveolar pattern among the alligatoroids *Alligator munensis* sp. nov. holotype DMR-BSL-2011-2 (**a**), *Allognatosuchus wartheni* YPM-PU 16989 (**b**), and *Navajosuchus mooki* AMNH 6780 (**c**). White dotted circles indicate the fourth maxillary alveolus. Series of small maxillary alveoli posterior to the fourth alveolus are indicated with the blue dotted circles, whereas enlarged maxillary alveoli are indicated by yellow dotted circles. Note that *Allognatosuchus wartheni* has extremely hypertrophied alveoli. Abbreviation: m4, fourth maxillary alveolus. Scale bar: 1cm. Figure generated using Adobe Illustrator CC.

3. References

Chaimanee, Y., Suteethorn, V., Jintasakul, P., Vidthayanon, C., Marandat, B., and Jaeger, J. J. (2004) A new orang-utan relative from the Late Miocene of Thailand. *Nature* 427, 439–441.

Claude, J., Naksri, W., Boonchai, N., Buffetaut, E., Duangkrayom, J., Laojumpon, C., Jintasakul, P., Lauprasert, K., Martin, J., Suteethorn, V., and Tong, H. (2011). Neogene reptiles of northeastern Thailand and their paleogeographical significance. *Ann. de Paléontol*. 97, 113–131.

Department of Mineral Resources (2007). Geological map of Nakhon Ratchasima Province. Geological map by Province.

Duval, M., Fang, F., Suraprasit, K., Jaeger, J-J., Benammi, M., Chaimanee, Y., Cibanal, J.I., and Grun, R. (2019). Direct ESR dating of the Pleistocene vertebrate assemblage from Khok Sung locality, Nakhon Ratchasima province, northeastern Thailand. *Palaeontol. Electron*. 22, 1–25.

Esposito, M., Chaimanee, Y., Jaeger, J.J., and Reyss, J.L. (1998). Datation des concrétions carbonatées de la Grotte du Serpent (Thaïlande) par la méthode Th/U. C. R. *Acad. Sci. Ser. IIA* 326, 603–608.

Esposito, M., Reyss, J.L., Chaimanee, Y., and Jaeger, J.J. (2002). U-series Dating of Fossil Teeth and Carbonates from Snake Cave, Thailand. *J. Archaeol. Sci*. 29, 341–349.

Suraprasit, K., Jaeger, J.-J., Chaimanee, Y., Benammi, M., Chavasseau, O., Yamee, C., Tian, P., Panha, S. (2015). A complete skull of Crocuta crocuta ultima indicates a late Middle Pleistocene age for the Khok Sung (northeastern Thailand) vertebrate fauna. *Quat. Int*. 374, 34–45.

Suraprasit K, Jaeger JJ, Chaimanee Y, Chavasseau O, Yamee C, Tian P, Panha S. (2016). The Middle Pleistocene Vertebrate Fauna from Khok Sung (Nakhon Ratchasima, Thailand): Biochronological and Paleobiogeographical Implications. *ZooKeys* 613, 1–157.

Suraprasit, K., Jaeger, J.-J., Chaimanee, Y., Sucharit, C. (2021). Taxonomic reassessment of large mammals from the Pleistocene Homo-bearing site of Tham Wiman Nakin (Northeast Thailand): relevance for faunal patterns in mainland Southeast Asia. *Quat. int.* 603, 90–112.
